# Investigating DNA supercoiling in eukaryotic genomes

**DOI:** 10.1093/bfgp/elx007

**Published:** 2017-04-24

**Authors:** Samuel Corless, Nick Gilbert

**Keywords:** DNA supercoiling, eukaryotes, chromatin, psoralen

## Abstract

Supercoiling is a fundamental property of DNA, generated by polymerases and other DNA-binding proteins as a consequence of separating/bending the DNA double helix. DNA supercoiling plays a key role in gene expression and genome organization, but has proved difficult to study in eukaryotes because of the large, complex and chromatinized genomes. Key approaches to study DNA supercoiling in eukaryotes are (1) centrifugation-based or electrophoresis-based techniques in which supercoiled plasmids extracted from eukaryotic cells form a compacted writhed structure that migrates at a rate proportional to the level of DNA supercoiling; (2) *in vivo* approaches based on the preferential intercalation of psoralen molecules into under-wound DNA. Here, we outline the principles behind these techniques and discuss key discoveries, which have confirmed the presence and functional potential of unconstrained DNA supercoiling in eukaryotic genomes.

## Introduction

**Figure 1:**
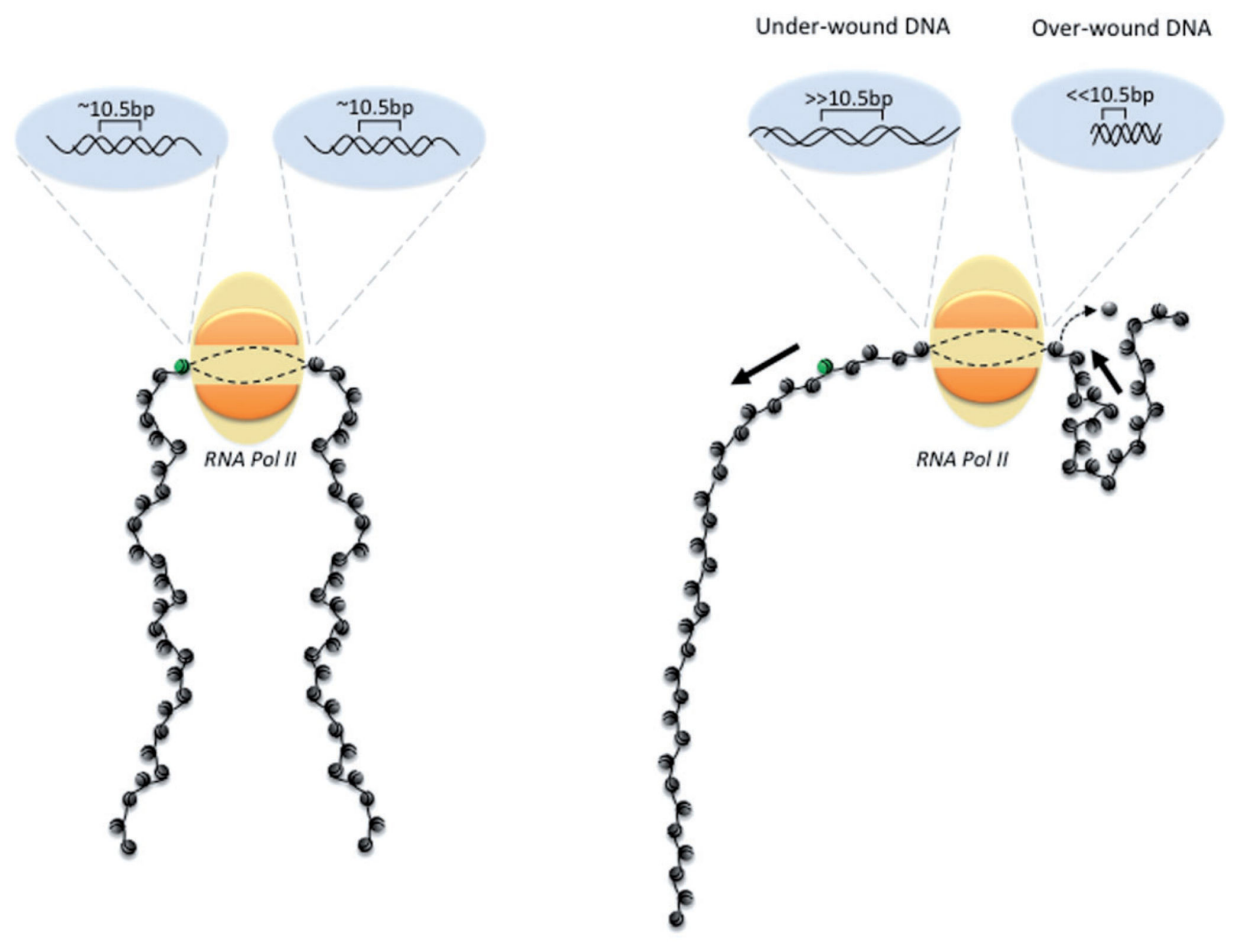
DNA supercoiling results in an over- or under-winding of the double helix. Representation of over-wound DNA ahead of and under-wound DNA behind the transcribing RNA polymerase complex. The green nucleosome indicates the movement of DNA through the RNA polymerase complex. The large complex size prevents rotation with the turn of the DNA helix and therefore generates supercoils via the twin-domain model. Over-wound DNA ahead of the polymerase complex destabilizes nucleosomes immediately ahead of the transcription machinery. (A colour version of this figure is available online at: https://academic.oup.com/bfg)

A fundamental component of genome packaging and regulation is DNA supercoiling, a transition in DNA structure from a relaxed double helix to one that is over- or under-wound (Figure 1, Box 1). Supercoils are generated by DNA-binding proteins as a consequence of bending, transcribing or replicating a length of DNA and are introduced by nucleosome core particles, DNA helicases and DNA/RNA polymerases [1]. For example, when the large RNA polymerase complex (>2 MDa) [2, 3] transcribes a region of chromatin, it cannot rotate with the tight helical path of DNA and therefore generates over-wound DNA ahead of the polymerase and under-wound DNA behind of the polymerase (Figure 1) [4]. This makes transcription a potent generator of supercoils, which introduce a rotational torque into the DNA helix [4, 5].

DNA supercoils can exist in an unconstrained state, where they are free to dissipate through the helix and transiently influence DNA structure, or they can be constrained within nucleoprotein complexes. For example, the nucleosome core particle constrains a single negative supercoil through structural distortion of the 147 bp of DNA wrapped around a histone octamer [6]. The free energy of unconstrained DNA supercoiling has the potential to influence key steps in gene regulation including the formation of an open promoter complex, transcription initiation, elongation and pausing [5, 7–9]. Much of this potential has been described *in vitro* or in prokaryotes, i.e. in systems that maintain the entire DNA in a strongly unconstrained under-wound state [10, 11]. On the contrary, eukaryotes constrain DNA supercoils in nucleosome core particles, and early studies concluded that no under-wound DNA was maintained in an unconstrained form in chromatinized genomes [11]. Mounting evidence refutes this idea [12–23], suggesting that eukaryotes instead maintain a more locus- or gene-specific enrichment for under-wound DNA, related to the specific regulation of transcriptionally active regions.

This review will outline the techniques that have pushed forward our understanding of the presence, maintenance and function of unconstrained DNA supercoiling in eukaryotic genomes.

## Centrifugation and electrophoresis as direct measures of DNA supercoiling

**Figure 2:**
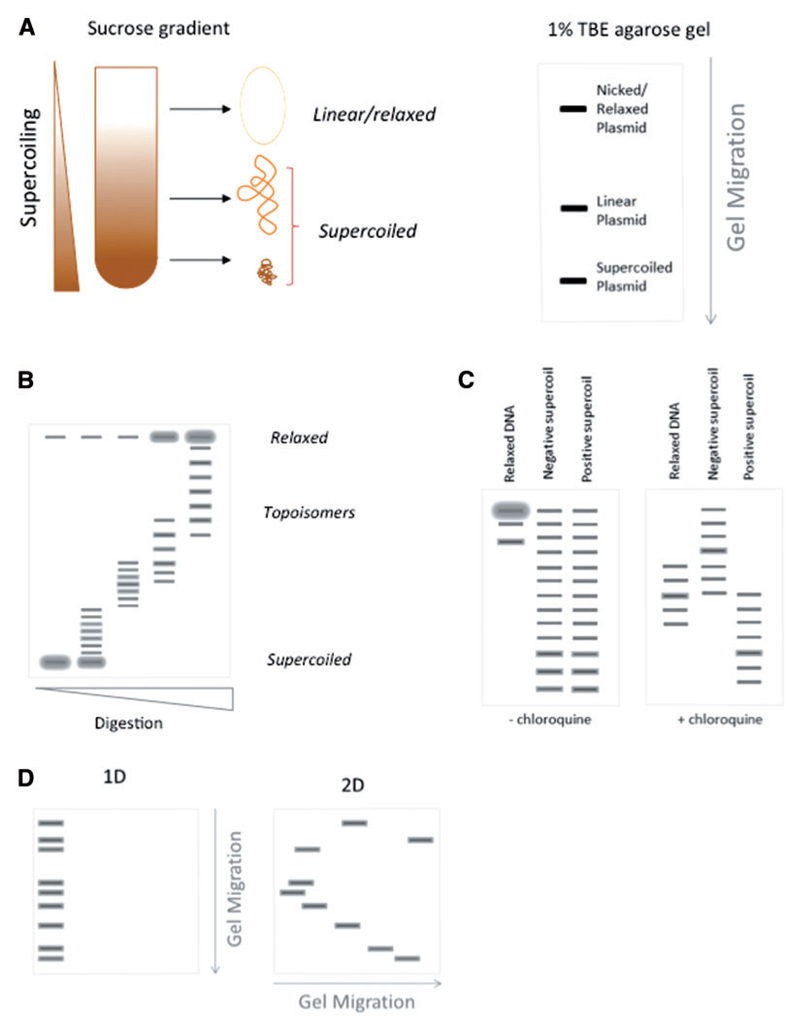
Centrifugation and electrophoresis to identify the supercoils present in circular DNA sequences. **(A)** Sucrose-gradient sedimentation and agarose gel electrophoresis differentiate DNA supercoil level based on the preferential migration of highly supercoiled/writhed molecules. **(B)** 1D agarose gel electrophoresis in the presence of low concentrations of an intercalator can differentiate topoisomers containing defined numbers of supercoils. **(C)** Chloroquine gels can be used to differentiate positively and negatively supercoiled DNA. **(D)** 2D agarose gel electrophoresis differentiates positive and negative supercoil topoisomers. (A colour version of this figure is available online at: https://academic.oup.com/bfg)

The basis for analysing DNA supercoiling by centrifugation or electrophoresis depends on the differential migration through a sucrose gradient or agarose matrix of molecules with the same molecular composition but different three-dimensional structures. In these techniques, supercoiling drives the DNA to adopt a compacted writhed structure that increases the sedimentation rate/electrophoretic mobility in a manner proportional to the level of supercoiling/writhe/compaction (Figure 2A). The beauty of these techniques lies in their simplicity—any difference in mobility of a purified circular DNA can only be explained by differences in DNA topology. On the other hand, these techniques are generally limited to study a single defined plasmid system per experiment and therefore lack the size and complexity of a eukaryotic genome. Despite these limitations, many of the key properties linking supercoiling to gene regulation have been established using these approaches, and they continue to remain a valuable tool.

### Approach

Early studies of DNA plasmids using ultracentrifugation approaches showed that different structures were present in a sample containing only plasmids of equal molecular weight [24–27]. The nature of this structural difference was determined by Vinograd *et al.* [28], who showed that a single-strand nick triggered the sedimentation of a single species, indicating that DNA normally had a constrained ‘twisted circular structure’. Using sucrose-gradient sedimentation, together with the DNA intercalator ethidium bromide, it was possible to accurately determine the number of supercoils within a plasmid DNA sample through a laborious titration approach [29]. Sucrose-gradient sedimentation proved highly informative for characterizing DNA supercoiling in plasmids, but has been largely superseded by simpler experimental approaches and is now rarely used for this purpose.

A more straightforward approach for analysing DNA supercoiling in closed circular plasmids is agarose gel electrophoresis. In a standard 1% TBE (Tris Borate EDTA) agarose gel without intercalating agent, DNA runs as three clear bands, which represent relaxed/nicked circular DNA, linear DNA and supercoiled DNA (Figure 2A) [30, 31]. The majority of supercoiled DNA will run as a single band, and this kind of assay can be useful when determining the total proportion of relaxed to supercoiled DNA [32]. To differentiate topoisomers with different levels of DNA supercoiling within the supercoiled template (Figure 2B), gels must be run in the presence of an intercalating agent—ethidium bromide or more commonly chloroquine [1]. For intercalating agents to bind the DNA needs to unwind, introducing positive supercoils that change the electrophoretic mobility of the DNA molecules. Using this property, chloroquine can be used to distinguish positively from negatively supercoiled DNA, by changing the supercoil density and electrophoretic mobility of the relaxed, positively and negatively supercoiled templates relative to one another (Figure 2C). However, when studying DNA from eukaryotic sources, it is not normally necessary to account for positively supercoiled DNA, as nucleosomes constrain negative supercoils, and deproteinization before agarose gel electrophoresis strongly biases plasmid DNA towards negative supercoils. Therefore, for most applications relevant to the understanding of DNA supercoiling in eukaryotes, one-dimensional (1D) gel electrophoresis without chloroquine titration is sufficient to characterize the relevant features.

An additional approach to study positive and negative DNA supercoiling uses a refined two-dimensional (2D) agarose gel electrophoresis technique (Figure 2D). In this approach, supercoiled plasmid DNA samples are first run in low percentage agarose (~0.4% is typical) in the presence/absence of intercalating agent, followed by a second electrophoresis in higher percentage agarose (~1% typical) at 90° to the first and again in the presence/absence of intercalator (Figure 2D). This permits the resolution of both positively and negatively supercoiled topoisomers and yields more detailed information about plasmid topology. For example, in a recent study, the 2D agarose gel electrophoresis protocol was developed further to allow the differentiation of different types of DNA knots, distinct DNA catenanes and other DNA structures [33].

### Discoveries and applications

#### Eukaryotic transcription is more efficient on a supercoiled DNA template

To determine the influence of DNA topology on transcription in eukaryotes, a number of studies have established the relationship between transcription and the supercoil status of a transfected plasmid, using 1D gel electrophoresis. In every case, intact circular DNA is the preferred substrate for transcription when compared with a linear template [7–9, 31–35]. Chromatin forms on both linear and circular plasmid DNA, but supercoiling can only form in intact circular DNA (unless the DNA is extremely long and/or tethered). Furthermore, increased expression can be seen in supercoiled DNA before the establishment of chromatin on transfected plasmids *in vivo* [31]. Together, these data support an important role for DNA supercoiling in eukaryotic gene expression.

#### Unconstrained DNA supercoils can be maintained in eukaryotes

The importance of DNA supercoiling for the expression of transfected plasmids does not necessitate that this supercoiling is unconstrained within the DNA, and it may instead be protein associated. To determine whether DNA supercoils can exist in an unconstrained state, plasmids have been transfected into eukaryotic cells and supercoiling carefully assayed by 1D and 2D agarose gel electrophoresis. Importantly, the supercoils present in transfected plasmids are not completely accounted for by bound nucleosomes, supporting the presence of non-protein-associated (unconstrained) DNA supercoils in a chromatinized template. For example, Ryoji and Worcel [31] show that chromatin assembly occurs within 10 min after DNA injection into frog oocytes, but supercoiling continues to increase up to 330 min, and that gene expression is related to the degree of supercoiling and not the extent of chromatinization. More recently, Kouzine *et al.* [36] used a stable plasmid system in human cells to show that the DNA between inducible divergent promoters becomes more negatively supercoiled when the genes are active. This increase in negative supercoiling alters the structure of a DNA sequence element previously shown to denature in the presence of unconstrained DNA supercoils, the far upstream element (FUSE) of c-Myc, indicating that the energy of DNA supercoiling is unconstrained in this situation. Together, these data confirm that unconstrained DNA supercoils can be generated in plasmids within eukaryotic cells.

#### Transcription initiation at eukaryotic promoters is enhanced by DNA supercoiling

The typical model of gene regulation by DNA supercoiling at promoters is that under-wound DNA facilitates the formation of an active promoter region and promotes transcription initiation [16, 19, 34, 37]. To identify whether unconstrained DNA supercoils can regulate gene expression through this mechanism in eukaryotes, *in vitro* studies of supercoiled, nicked, relaxed and linear plasmids have been performed for a small number of gene promoters. Using agarose gel electrophoresis, Mizutani *et al.* [32] characterized the supercoil state of plasmid DNA and compared this with the corresponding transcription level. In some, but not all, cases, gene expression was significantly enriched in the presence of unconstrained negative DNA supercoiling. However, the panel of promoters assayed through this approach is so far extremely limited, and there is scope for a high-throughput analysis of promoter DNA sequence to establish the properties that determine supercoil sensitivity. Furthermore, there is just a single study that characterizes the mechanism by which DNA supercoiling influences gene regulation and shows that supercoiling promotes transcription initiation and not the transition to an elongation complex or subsequent elongation [8]. Recent advances permit the chemical synthesis of any desired DNA sequence, and it is an achievable prospect to generate a high-throughput approach to analyse the *in vitro* sensitivity of hundreds of gene promoters to DNA supercoiling. By comparing DNA supercoiling data, generated through agarose gel electrophoresis-based approaches, with transcription data, a wealth of information could be generated for the interpretation of supercoiling within eukaryotic genomes.

#### DNA structure is influenced by unconstrained supercoiling in eukaryotic chromatin

Negative supercoils promote the formation of DNA melting and non-B DNA structures including Z-DNA, G-quadruplexes, cruciforms and R-loops [1, 38, 39]. Experimental evidence increasingly supports the presence of these alternative structures *in vivo* [40–46], but their relationship with DNA supercoiling *in vivo* remains largely uncharacterized. Work in the Levens laboratory [14, 36, 47–49] has characterized the FUSE DNA element, which displays supercoil-dependent melting and regulates binding of the FUSE binding protein and FUSE interacting repressor (FIR). The supercoiling-dependent structural transition of FUSE was determined *in vitro* by 2D agarose gel electrophoresis, identifying a level of supercoiling where the compaction through writhe formed a plateau because of the localized melting of DNA [48], and this melting has been confirmed *in vivo* in an episomal plasmid system [36]. Many other alternative structures have been characterized *in vitro* by agarose gel electrophoresis of plasmid systems [50–55], but little is known of their capacity to form *in vivo* and whether their formation promotes the binding of regulatory proteins. To further characterize the relationship between DNA supercoiling and alternative DNA structure in chromatinized DNA in eukaryotes, 1D and 2D agarose gel electrophoresis of isolated plasmids will continue to be a valuable tool.

#### Characterizing properties of DNA supercoiling in replication

In addition to influencing gene regulation, DNA supercoiling has been proposed to be important for other aspects of genome structure including genome packaging before cell division. Several studies have suggested that supercoiling promotes the separation of interlinked DNA strands following DNA replication in prokaryotes, a process called decatenation [56, 57]. Adapting 1D and 2D gel electrophoresis approaches to study yeast centromeric plasmids showed that positive supercoiling, generated by mitotic spindles and condensin, maximizes DNA decatenation activity by topoisomerase II and may drive full decatenation of a eukaryotic genome [30, 58]. Importantly, in wild-type conditions, the yeast plasmids never become positively supercoiled, rather it is the generation of positive supercoils and their subsequent removal that decatenates the genome, giving no net change in DNA supercoil level.

### Perspective

Centrifugation and agarose gel electrophoresis approaches have determined some of the key properties of DNA supercoiling in eukaryotes, using transfected or stable plasmid systems. A major limitation of these approaches is that they do not necessarily reflect the properties found in eukaryotic chromosomes, which are orders of magnitude larger, are regulated by distinct mechanisms and have evolved specifically to deal with the topological issues most prevalent in their cell type. Perhaps for this reason, using plasmids and electrophoresis to characterize DNA supercoiling *in vivo* has, with notable exceptions [30, 36, 59–61], been less prevalent in the literature in recent years. However, there remains valuable insight to be achieved using these techniques if we are to understand the mechanisms linking supercoiling to genome regulation *in vivo.*

## Psoralen as a molecular probe for DNA supercoiling

To measure DNA supercoiling in the normal chromosomes of eukaryotic cells, the intercalating agent psoralen has been used as a molecular probe for under-wound DNA supercoils (see Box 1 for definition of under-wound). Psoralen molecules preferentially intercalate into under-wound DNA and can form stable cross-links to DNA when exposed to ultraviolet (UV) irradiation at 365 nm [62]. The measure of supercoiling is more indirect than that of agarose gel electrophoresis, but the capacity to probe-specific loci is invaluable. The properties of DNA supercoiling within eukaryotic genomes have been largely characterized using psoralen-based methods, and further development will help define the function of unconstrained DNA supercoiling *in vivo*.

### Approach

The psoralen derivative 4,5’,8-trimethylpsoralen (TMP) is a cell permeable planar molecule that intercalates between base pairs in the DNA double helix and forms stable photo-cross-links with pyrimidine nucleotides on exposure to 365 nm UV light [62]. Importantly, the preferential intercalation of TMP into under-wound DNA has been established in both naked and chromatinized DNA and is therefore applicable for characterizing supercoiling in eukaryotic cells [13]. TMP can form mono-adducts or inter-strand cross-links with the DNA double helix, with ∼15 mono-adducts forming for every inter-strand cross-link [63], and can be chemically modified to include a biotin tag. Using these properties, various experimental methods have been developed to identify the localization of under-wound DNA within eukaryotic genomes.

#### Denaturing approach to enrich for inter-strand cross-links

**Figure 3:**
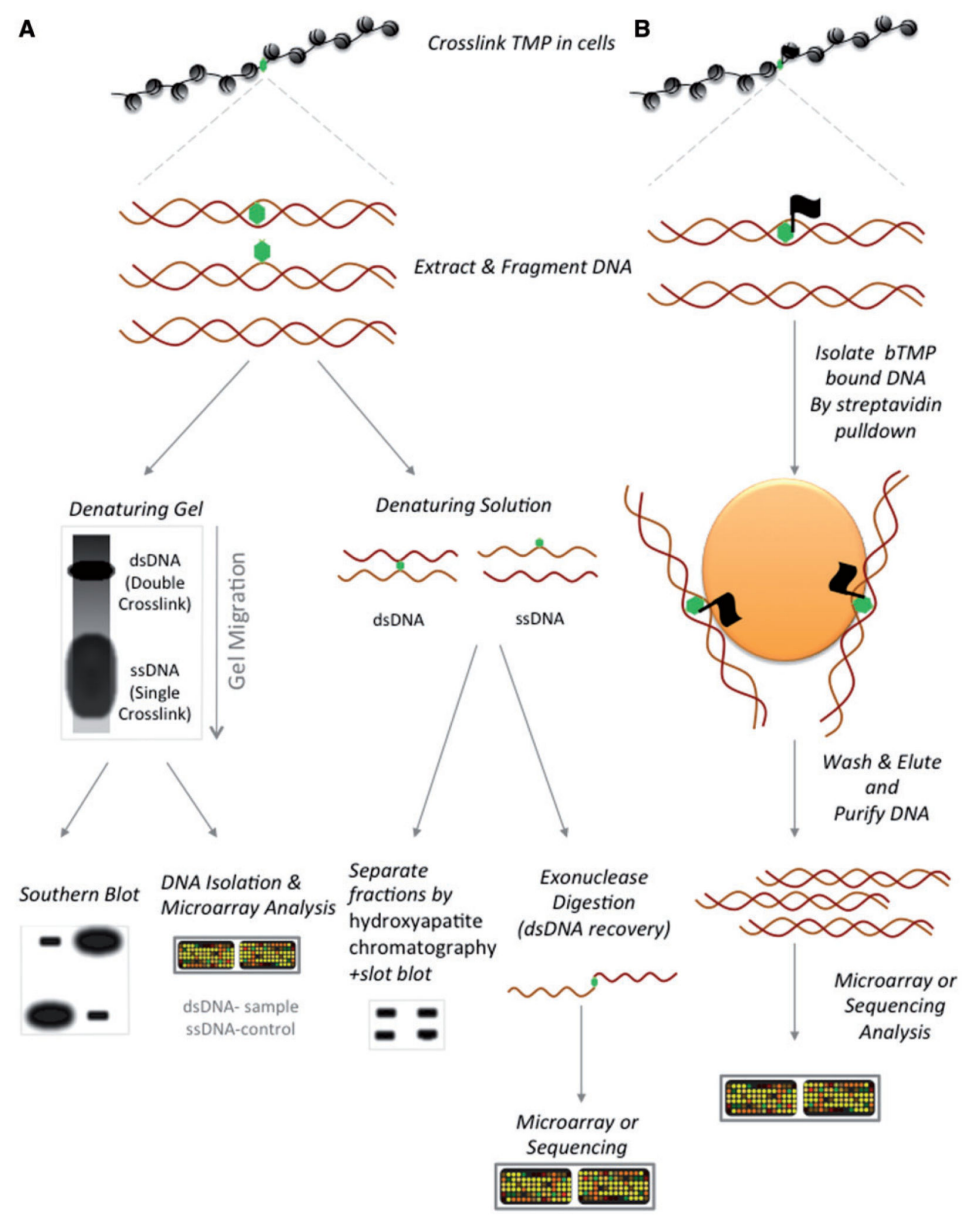
Psoralen-based approaches identify enrichments of under-wound DNA in eukaryotic genomes *in vivo*. **(A)** Approaches taking advantage of inter-strand cross-links formed by a proportion of covalently linked psoralen molecules. Denaturing gel/solution followed by electrophoresis/hydroxyapatite chromatography/exonuclease digestion permits the separation of DNA molecules with an inter-strand cross-link compared with those with no cross-links or a psoralen mono-adduct. Enrichment for inter-strand cross-links at particular loci is then assayed by Southern blot, slot blot, microarray or sequencing. **(B)** Biotin-psoralen pull-down approaches enrich for psoralen-bound DNA for analysis by microarray or sequencing. (A colour version of this figure is available online at: https://academic.oup.com/bfg)

One way to differentiate under-wound regions that bind TMP takes advantage of the capacity of TMP to form inter-strand cross-links between the two strands of the double helix. By incubating cells in the presence of TMP and cross-linking the drug to DNA by UV irradiation, a portion of the covalently attached TMP molecules will form inter-strand cross-links that stabilize the DNA double helix. Using this property, two methods have been developed to analyse the distribution of under-wound DNA.,

In the first method fragmented DNA samples are processed by denaturing gel electrophoresis (Figure 3A), which causes the DNA to run as two fractions—a higher molecular weight band of double-stranded DNA (dsDNA) maintained by TMP cross-links and a lower molecular weight single-stranded DNA (ssDNA) band containing DNA with TMP mono-adducts or no TMP bound. Sequences can then be analysed by Southern blot to determine whether a specific sequence (e.g. gene transcription start site) is enriched for under-wound DNA supercoiling by determining the relative enrichment of the DNA probe in the dsDNA compared with the ssDNA fraction [15, 20, 21]. A more recent adaptation of this technique analysed isolated dsDNA and ssDNA regions from the agarose gel using microarray [14].

In a second method, fragmented DNA samples are processed in solution to enrich for TMP-bound dsDNA (Figure 3A). Early studies used hydroxyapatite chromatography to separate dsDNA and ssDNA followed by a slot-blot approach [18, 19], which gave results equivalent to those of the Southern blot procedure. More recently, several groups have used exonuclease digestion of denatured DNA to enrich for DNA with inter-strand cross-links [13, 17]. In this approach, ssDNA is fully denatured, whereas DNA with inter-strand cross-links only partially denatures, maintaining a TMP bridge between the two strands. Exonucleases degrade ssDNA entirely, but are interrupted by the TMP inter-strand cross-link to leave 3′ ssDNA overhangs. These DNA samples are isolated and analysed by microarray or deep sequencing to give the distribution of inter-strand cross-links, similar to those described for the denaturing gel-based approach.

#### Pull-down approach to enrich for under-wound DNA

A second way for enriching TMP-bound DNA is to redesign the molecule to include a molecular tag that allows purification of the under-wound DNA using a pull-down approach (Figure 3B). The major advantage of this technique is that it enriches for both inter-strand cross-links and the more highly abundant mono-adduct TMP molecules [63]. Therefore, TMP can sample differences in DNA supercoiling by detecting all bound TMP molecules, rather than the minority of TMP molecules that form inter-strand cross-links (around 1 of 15 TMP molecules). In our laboratory, we biotinylated the TMP (bTMP) molecule following the structure devised by Saffran *et al.* [64]. Cells were incubated with bTMP before cross-linking with UV irradiation, followed by DNA fragmentation, DNA purification and the enrichment for under-wound DNA by pull-down with streptavidin beads followed by hybridization to microarrays [16]. A similar approach was subsequently used by Anders *et al.* [12] using a bTMP to enrich for TMP-bound DNA and analysis by next-generation sequencing.

#### Immunofluorescence approaches to visualize under-wound DNA supercoiling

In addition to sequence-based approaches for mapping DNA supercoiling *in vivo*, bTMP has been used to visualize the distribution of DNA supercoils in immunofluorescence-based techniques. To visualize under-wound DNA in human cells [16] and in the *Drosophila* polytene chromosome [15], bTMP was cross-linked into the DNA, followed by sample fixation, the addition of a streptavidin-labelled fluorescent probe and visualization by fluorescence microscopy.

#### Sequence considerations and controls for TMP distribution analysis

In all the methods discussed above, it is important to consider properties of TMP in the design of experiments and interpretation of results [65], the most important of which is to consider the potential influence of sequence bias on TMP binding. Attempts to determine the properties of TMP sequence bias have identified that the molecule shows no sequence bias when binding DNA [66], but that the formation of UV cross-links has a strong preference towards thymidine nucleotides. Furthermore, the influence of local sequence on TMP cross-link frequency is complex and unpredictable, with a preference for 5′TA over 5′AT, a strong influence of flanking bases up to 3 bp either side and potential long-range effects over tens of base pairs [66–68]. The clear influence of local sequence context on TMP-DNA cross-links suggests that sequence-dependent DNA helical structure is important for TMP binding. Therefore, it is important to differentiate under-wound DNA supercoil distribution from this complex sequence bias, and the simplest way to do this is to compare two conditions in which the sequence bias is not expected to change. For example, the addition of a transcription inhibitor allows the identification of under-wound DNA that is generated by active transcription [14–16].

To give an absolute distribution of supercoiling, the DNA can be nicked, either chemically [15, 16] or through X-ray irradiation [18, 19], to dissipate supercoils and provide a base line for relative enrichment of under-wound DNA. Another baseline from which to determine the relative enrichment of under-wound DNA is to compare TMP distribution on genomic DNA with that in cells [13, 16]. This has the caveat of comparing a chromatinized template with a non-chromatinized template, but experiments in our laboratory suggest that the distribution of bTMP in genomic DNA and bleomycin-treated cells is broadly similar [16]. Finally, a selection of other inhibitors/conditions have been used to tease apart differences in DNA supercoiling independent of sequence, including heat shock [15, 20], topoisomerase knock out [13] and topoisomerase inhibition [16]. Together, these results by Southern blot, microarray and immunofluorescence assays, all support difference in DNA supercoiling in eukaryotic genomes as measured by TMP.

### Discoveries and applications

#### Unconstrained DNA supercoiling is present in eukaryotes

Early studies assaying whole-genome TMP binding in bacteria, *Drosophila* and human concluded that prokaryotes maintain their genome in a strongly under-wound state whereas, at the limit of their detection methods, eukaryotic DNA is not maintained in a globally unconstrained under-wound state [11]. Using a TMP cross-link followed by denaturation approach to perform a more focused analysis of DNA supercoiling at gene promoters and enhancers, several groups identified that unconstrained DNA supercoiling is present at active genes in human, fly and hamster cells [18–20]. For example, Ljungman and Hanawalt [18] show that the 5′ ends of human *DHFR* and ribosomal DNA genes are enriched for TMP inter-strand cross-links under normal conditions, but not when the DNA is nicked by X-ray irradiation.

These early studies supported the idea of ‘micro-domains’ of under-wound DNA supercoiling present in a genome that was almost entirely devoid of unconstrained supercoiling. Immunofluoresence data in *Drosophila* polytene chromosome transformed this view by demonstrating that regions of under-wound DNA are prevalent throughout genomes and are strongly correlated with transcriptionally active regions [15]. Further, characterization of under-wound domains identified that they are lost on nicking the genome and following transcription inhibition. Similarly, our laboratory identified in human cells that under-wound DNA supercoiling is present throughout the nucleus and that the bTMP signal on bleomycin treatment to nick the DNA is reduced [16].

To map the under-wound DNA, which was by then known to be prevalent in eukaryotic genomes, several groups adopted an approach where TMP-bound DNA was enriched and hybridized to microarrays tiling regions [13, 14, 16] of the genome or analysed by next-generation sequencing [12, 17]. The first study to use this approach compared wild-type and topoisomerase mutant yeast, showing that domains of differential supercoiling exist between mutant and wild-type strains [13]. In our laboratory, we applied a similar technique in human cells and identified ~100 kb domains that are relatively under-wound or over-wound [16]. Furthermore, we identified a general enrichment for under-wound DNA at promoters, as shown for a few key examples in previous studies. This promoter enrichment has now been confirmed in a number of further studies [12, 14, 17].

Together, these data provide strong evidence that under-wound DNA supercoiling is present in the genomes of eukaryotes as both large-scale domains and a more focused local enrichment such as at gene promoters.

#### Under-wound DNA is associated with active transcription *in vivo*

Under-wound DNA is associated with transcription initiation *in vitro* and in prokaryotes, and experiments using TMP have now demonstrated an association in eukaryotes. For example, Jupe *et al.* [20] showed in *Drosophila* that TMP inter-strand cross-links are enriched at active 18S ribosomal RNA genes and at heat shock genes following stimulation, but not at a nearby downstream region. Developing this idea further, Matsumoto and Hirose [15] show by immunofluorescence that a heat shock locus in *Drosophila* exhibits high levels of under-wound DNA supercoiling after stimulation, unless the DNA is nicked or transcription is inhibited. Similar observations in hamster [19] and human [14, 16] support this link, and work in our laboratory has demonstrated that large-scale domains and local-enrichment at promoters are substantially rearranged on transcription inhibition. Together, these data support a relationship in which active transcription generates a local enrichment of under-wound DNA supercoiling.

### Perspective

#### Implications and future directions

The identification of unconstrained DNA supercoiling in higher eukaryotes transforms our understanding of the potential role that DNA molecules can play in facilitating and signalling their own transcription events. Using centrifugation/electrophoresis and psoralen-based approaches over the past 35 years, the presence and distribution of DNA supercoiling *in vivo* in eukaryotes have been established, and recent advances have shown that unconstrained under-wound DNA is a general property of actively transcribed promoters and large-scale domains. Furthermore, these DNA structures are transient and can be disrupted by nicking the genome, inhibiting transcription or suppressing topoisomerase activity. Future work must characterize in detail the introduction, maintenance and influence of DNA supercoiling on eukaryotic genome regulation.

#### Introduction of DNA supercoils

As far as we are aware, transcription generates most of the supercoiling in eukaryotic DNA, via the twin-domain model in which DNA is over-wound ahead of the advancing polymerase and under-wound behind (Figure 1) [4]. This is in contrast to prokaryotes, which have specific DNA gyrase enzymes that can introduce under-wound DNA supercoils [69]. Therefore, to better understand the distribution of DNA supercoils *in vivo*, we must also know where transcription in the genome occurs. In the past decade, our understanding of the distribution of transcription *in vivo* has been transformed by techniques that precisely map nascent transcription including GRO-seq [70], PRO-seq [71], Start-seq [72], etc. These techniques have demonstrated that the majority of transcription is noncoding, with abortive transcripts most common at the promoter regions of genes. Consequently, DNA supercoiling must be highest in these regions and may then dissipate to have local-scale and domain-scale influence. A recent model has proposed that the coupling of transcription to DNA supercoiling can recapitulate experimental observations, including transcription bursts and the upregulation of divergent or bidirectional genes [73]. This model predicts how gene orientation and the action of topoisomerase enzymes will influence the co-regulation of neighbouring genes and an important future goal is to test the predictions of this model *in vivo*. One key parameter required to further understand these properties is to determine how supercoils introduced into the genome by a specific transcription event dissipate from their origin *in vivo* and influence steady-state DNA supercoil distribution locally and over large-scale domains.

#### Maintenance of DNA supercoils

Psoralen studies in higher eukaryotes have identified that DNA supercoiling is maintained *in vivo* by a balance of transcription and topoisomerase activities, and that perturbation of either can cause promoter-scale and large-scale changes in DNA supercoil distribution. However, the mechanism linking this balance in activity remains unknown. In theory, eukaryotic topoisomerase proteins should remove both over-wound and under-wound DNA supercoils with similar efficiency to leave a net state of relaxed DNA [74, 75]. How under-wound DNA is maintained at the expense of over-wound DNA remains unknown, although it is tempting to speculate that mechanisms exist to preferentially remove over-wound supercoils to prevent the transcription machinery from pausing/stalling [5, 76]. Furthermore, the relative influence of topoisomerase I and II on the maintenance of DNA supercoils is not well characterized. Chromatin immunoprecipitation studies suggest a relationship between transcribed regions and topoisomerase I [16, 77–79], and protein studies suggest that RNA polymerase II and topoisomerase I interact [80], although a recent study has demonstrated that topoisomerase I activity is strongly depleted over gene promoters, despite high levels of associated protein [79]. Similarly topoisomerase II is enriched at gene promoters [81–84], and it has been demonstrated that dsDNA breaks generated by topoisomerase II are required for the regulated transcription of certain genes [85]. Future studies must address how topoisomerase activity relates to DNA supercoils *in vivo* and address the relationship between topoisomerase activity at specific loci and the influence on DNA supercoil distribution.

#### Influence of DNA supercoils *in vivo*

DNA supercoils have broad influence on DNA structure, and the identification of unconstrained supercoils in eukaryotes opens a huge field of regulatory potential. In recent years, a number of alternative DNA structures stabilized by under-wound DNA supercoils have been identified in higher eukaryotes *in vivo*, including R-loops, G-quadruplexes, cruciforms, Z-DNA and ssDNA. Proteins including transcription factors have been shown to specifically associate with such DNA structures [40, 51, 52, 86], as well as more subtle differences in DNA structure such as the transition from a B-form to an A-form helix and the localized under-winding of DNA [87, 88]. In these cases, it has not been established whether the change in DNA structure causes or is a consequence of protein binding, although there is an increasing evidence supporting a role for DNA structure in this process [89]. Furthermore, the direct association between changes in unconstrained DNA supercoiling and transcription factor binding has only been demonstrated for one example, the FUSE interacting protein and FIRs at the supercoil-sensitive FUSE DNA element [36]. In future work, investigators must systematically test other supercoil-sensitive elements for (a) the formation of alternative DNA structures as a result of DNA supercoiling and (b) the specific binding of regulatory proteins to these alternative DNA structure and the specific regulation of transcription/replication as a result. It is noteworthy that DNA sequence motifs for alternative DNA structures are highly enriched and evolutionarily conserved at gene promoters and human replication origins [90], further supporting a potential functional relevance for supercoil-dependent DNA structural transitions. Identifying whether these structures are a general mechanism for real-time signalling of ongoing transcription, and function to enhance/suppress future transcription is an essential next step for the DNA supercoil field.

In addition to altering the helical structure of DNA, supercoiling can introduce a rotational torque into the DNA, which facilitates the formation of the pre-initiation complex and subsequent gene expression at specific eukaryotic genes *in vivo* [8]. By this mechanism, supercoils generated by the transcription of one gene could dissipate through the DNA and influence transcription from the promoters of neighbouring genes [73], and supercoils generated by abortive transcription could facilitate full-length gene expression by priming the DNA structure of a promoter region [91]. Whether under-wound DNA supercoiling alters DNA structure directly, or provides the energy for proteins to do so, is unknown. Furthermore, the influence of under-wound DNA supercoiling on different eukaryotic promoters has not been widely tested, with a single *in vitro* study reporting increased transcription in two of three promoters [32]. Recent work has shown that eukaryotic gene promoters are generally under-wound [12, 14, 16, 17], particularly when active, and it is now important to establish the features of promoters *in vivo* that confer supercoil sensitivity. Only with this knowledge, can we begin to understand how domains of DNA supercoiling influence the expression properties of the gene promoters contained within them.

## Improved methods for detecting DNA supercoiling *in vivo*

In addition to using centrifugation, electrophoresis and psoralen-based approaches to address many of the outstanding questions in the field of DNA supercoiling, it is essential that future work identifies new approaches and methodologies for probing the influence of DNA supercoiling in eukaryotes *in vivo*.

Recent work using gel electrophoresis, a field that is >40 years old, demonstrate that exciting technical and biological questions continue to be addressed using these approaches [30, 33, 36, 48, 58]. Despite the inherent limitation of using a defined circular plasmid system, which does not represent the majority of eukaryotic DNA, gel electrophoresis approaches remain key for providing mechanistic insight for the role of DNA supercoiling *in vivo*. Future work in this field is mostly limited by the imagination and technical capacity required to elicit complex and generally applicable characteristics using this relatively simple system. A good starting point is the development of new independently replicating plasmid systems that address specific properties of DNA supercoiling *in vivo*, similar to recent studies of centromeric sequence or supercoil-sensitive sequences in eukaryotic cells [30, 36, 48, 59]. Using similar approaches, many outstanding questions could be addressed including the role of supercoiling in gene promoter regulation, transcription factor binding and alternative DNA structure formation *in vivo*.

In contrast to gel electrophoresis approaches, in which changes in DNA migration can only be attributed to changes in DNA structure, psoralen-based approaches suffer the limitations inherent to a chemical probe of genome structure. Psoralen has a complex sequence specificity and may show some preference for more accessible chromatin regions (although Kouzine *et al*. [14] provide data indicating this is not the case). Furthermore, it is not well characterized how psoralen binds non-B form DNA structures. While these issues are not unique to psoralen, for example the major chemical probe of chromatin structure is formaldehyde, which has strong DNA and peptide sequence bias (binding only guanines and lysines [92]), it is an important consideration. To reduce the influence of known and unknown psoralen bias on the interpretation of DNA supercoiling distribution, distributions were identified in cells under different conditions including on genomic DNA, with bleomycin treatment and with transcription/topoisomerase inhibition. For greater confidence in interpreting the properties of DNA supercoiling *in vivo*, future studies must aim to identify alternative probes for DNA supercoiling, taking advantage of features in addition to the increased capacity for the intercalation of planar molecules. For example, minor groove binders such as netropsin bind into the DNA and induce changes in DNA supercoiling that suggest these molecules could be used to probe over-wound DNA [93]. Other probes for DNA structure could include producing synthetic proteins, which preferentially bind supercoiled DNA, for example by using the ‘supercoiled DNA-recognition domain’ of LEDGF/p75 [94]. These molecular probes will supplement current and future experimental observations determined using psoralen, to elucidate the presence and function of DNA supercoiling in the genomes of eukaryotes.

## Summary

The presence of unconstrained DNA supercoiling is now well established in the chromatinized genomes of higher eukaryotes. The presence and characterization of these unconstrained supercoils have been identified using centrifugation, electrophoretic and psoralen-based approaches. Current and future work must adapt these techniques alongside cutting-edge developments in nascent RNA sequencing and alternative DNA structure mapping. Furthermore, novel chemical probes are required to corroborate observations with psoralen and the supercoil-sensitive FUSE sequence element. Together, these techniques will bring forward a new understanding for the role of DNA structure in signalling its own transcription and facilitating future transcription events in eukaryotic cells through DNA supercoiling.

## Box 1 Defining supercoiling in DNA

There are several ways to define the distribution of supercoiling in DNA, each of which is particularly suited to a certain theoretical or experimental situation. When describing supercoils in a closed circular piece of DNA, it is possible to discuss absolute differences in supercoiling in terms of linking number (Lk), which is the number of times one strand of the DNA crosses over the other in a closed circular plasmid. Lk_0_ represents the Lk of relaxed DNA, while negatively supercoiled DNA has an Lk less than the Lk_0_ (i.e. one strand crosses the other fewer times), and positively supercoiled DNA has an Lk greater than Lk_0_ (i.e. one strand crosses the other more times). The transition from a relaxed (Lk_0_) to a supercoiled (Lk ≠ 0) double helix requires a transition in DNA structure, which can be manifest as a change in the number of turns of the helix per nucleotide (twist) (negative <10.5 bp/turn, positive >10.5 bp per turn) and/or in the formation of a coiled helix/superhelix (writhe) (discussed in detail in [1, 38]). DNA writhe induces the compaction of a DNA circle (Figure 2A), and the ratio of twist:writhe remains constant for a particular Lk in circular DNA *in vitro* [95]; therefore, DNA circles with different absolute levels of supercoiling can be distinguished based on changes in DNA structure. This forms the basis of centrifugation- and electrophoresis-based approaches for analysing DNA supercoiling, and in these experiments, absolute measurement using Lk is most appropriate.

This nomenclature is of less use in complex eukaryotic genomes where the boundaries of supercoil dissipation, the definition of ‘relaxed’ DNA and the relative importance of twist and writhe remain unclear (discussed further in [91]). In this situation, we use the terms over-wound and under- wound DNA (Figure 1), which gives an indication of the relative change in DNA supercoiling between conditions (e.g. transcription inhibition). This is useful in psoralen-based experiments where changes in relative distribution of drug indicate changes in relative distribution of DNA supercoiling, but not the absolute level of this change.

## Key Points

- DNA supercoiling is a fundamental component of genome packaging and regulation.
- In eukaryotes, the role of DNA supercoiling is poorly understood.
- Techniques to study DNA supercoiling include agarose gel electrophoresis and psoralen-based molecular probes.
- Using these approaches, unrestrained DNA supercoiling has been identified and mapped in eukaryotic genomes.
- Current and future work aims to understand the role of DNA in facilitating its own transcription through DNA supercoiling.
